# Pallidal deep brain stimulation for Parkinson's disease: indications for target selection and perceived outcomes at two centers

**DOI:** 10.1016/j.prdoa.2026.100513

**Published:** 2026-10-14

**Authors:** Nestor Beltre, Hikaru Kamo, Gabriele Bellini, Ilona Cazorla-Morales, Adolfo Ramirez-Zamora, Michael S. Okun, Coralie de Hemptinne, Alon Mogilner, Michael Pourfar

**Affiliations:** a NYU Langone Health, Department of Neurology, United States of America; b Hackensack Meridian School of Medicine, United States of America; c Department of Neurology, Fixel Institute for Neurological Diseases, University of Florida, Gainesville, Florida, United States of America; d Department of Neurology, Juntendo University School of Medicine, 2-1-1 Hongo, Bunkyo-ku, Tokyo, 113-8421, Japan; e NYU Langone Health, Department of Neurosurgery and Neurology, United States of America; f NYU Langone Health, Department of Neurosurgery, United States of America

**Keywords:** Parkinson's disease, Deep brain stimulation, Globus pallidus internus, Dyskinesia, Dystonia, Neuropsychiatric consideratoins, Target selection

## Abstract

We retrospectively reviewed Parkinson's disease patients undergoing GPi-DBS at two academic centers to characterize clinical rationale for target selection. GPi was most commonly selected for dyskinesia, followed by dystonia and neuropsychiatric considerations, with clinicians reporting sustained subjective improvement in hyperkinetic symptoms at one year.

Deep brain stimulation (DBS) is an established therapy for Parkinson's disease (PD) patients with medically refractory motor complications [1], [2]. The subthalamic nucleus (STN) and globus pallidus internus (GPi) are the two most utilized targets, each offering distinct advantages [3]. While randomized trials demonstrate comparable overall motor benefit, real-world target selection is frequently driven by symptom-specific considerations; GPi-DBS is commonly favored for patients with troublesome dyskinesia, dystonia, or concerns regarding cognitive or psychiatric vulnerability [4], [5], [6]. However, the clinical rationale guiding GPi selection in routine practice has not been completely characterized.

We performed a retrospective chart review of PD patients who underwent GPi-DBS at two high-volume academic centers: New York University Langone Health (2013–2024) and the University of Florida Fixel Institute (2015–2021). Inclusion criteria were patients with Parkinson's disease who underwent GPi-DBS at either center; exclusion criteria were lack of documented clinical rationale for GPi target selection, no postoperative clinical assessment available (i.e., lost to follow-up before the assessment window), or GPi target selection based solely on anatomical considerations. In total, 143 patients underwent GPi-DBS and met inclusion criteria; 6 were excluded for the reasons above, leaving 137 patients in the final analysis.

The primary objective was to characterize the reasons clinicians selected GPi over STN in routine clinical practice. Secondary objectives were to describe subjective postoperative outcomes related to dyskinesia and dystonia, as documented in follow-up visits. Reasons for GPi selection were abstracted from multidisciplinary DBS conference notes and preoperative clinical documentation and categorized as dyskinesia, dystonia, and/or neuropsychiatric concerns. Multiple reasons could possibly be assigned to a single patient. Continuous outcomes were compared using Wilcoxon signed-rank tests; subjective categorical ratings were compared using Stuart–Maxwell tests for marginal homogeneity. All *p*-values were FDR-adjusted. Missing timepoints were excluded from the relevant comparison rather than imputed. Full statistical detail is provided in Supplementary Appendix S2.

The cohort had a median disease duration of 9 years and a median age at surgery of 65 years. Dyskinesia was the most common indication for GPi selection, and was present in 75.2% of patients (103), followed by dystonia in 49% (67). Neuropsychiatric considerations, including mild cognitive impairment, mood disorders, or behavioral concerns, were present in 30.7% of cases (42). These indications were frequently overlapping, reflecting the multifactorial nature of DBS target selection in advanced PD.

Postoperative clinical assessments were reviewed at approximately 6 months and 1 year following DBS surgery. Outcomes were assessed descriptively, focusing on clinician-documented and patient-reported impressions rather than formal rating scale–based change scores. Subjective change in dyskinesia and dystonia was classified as improved, unchanged, or worsened as relative to the preoperative state.

Subjective assessments based on both the objective clinical impression and patient self-report indicated that dystonia improved in 51 patients (60.7%), remained unchanged in 27 (32.1%), and worsened in 1 (1.2%). Similarly, dyskinesia improved in 56 patients (59.6%), remained unchanged in 33 (35.1%), and worsened in 3 (3.2%). At the 1-year visit, subjective assessments based on both the clinician's impression and the patient's report indicated that dystonia improved in 59 patients (89.4%) and remained unchanged in 7 (10.6%). Similarly, dyskinesia improved in 61 patients (78.2%) and remained unchanged in 16 (20.5%). These findings were consistent across both centers despite differences in programming practices and follow-up paradigms. Total electrical energy delivered and subjective dystonia and dyskinesia ratings at 6 months were directionally consistent with 1-year outcomes, with continued improvement observed between the two timepoints (Fig. 1, Panels C–E). Additionally, objective clinical outcomes, including UPDRS part III scores and levodopa-equivalent dosages demonstrated statistically significant improvements from baseline to 1-year post-op, and total energy delivered via the DBS system increased from 6 months to 1-year post operatively. This modest LEDD reduction is consistent with GPi-DBS's mechanism of action, which relies less on medication reduction for symptom control than STN-DBS. UPDRS Part III was assessed on-medication at both centers at baseline and 6 months; at 1 year, assessment continued on-medication at one center and shifted to off-medication at the other, per each center's standard protocol. Results are summarized in Fig. 1.

**Fig. 1:**
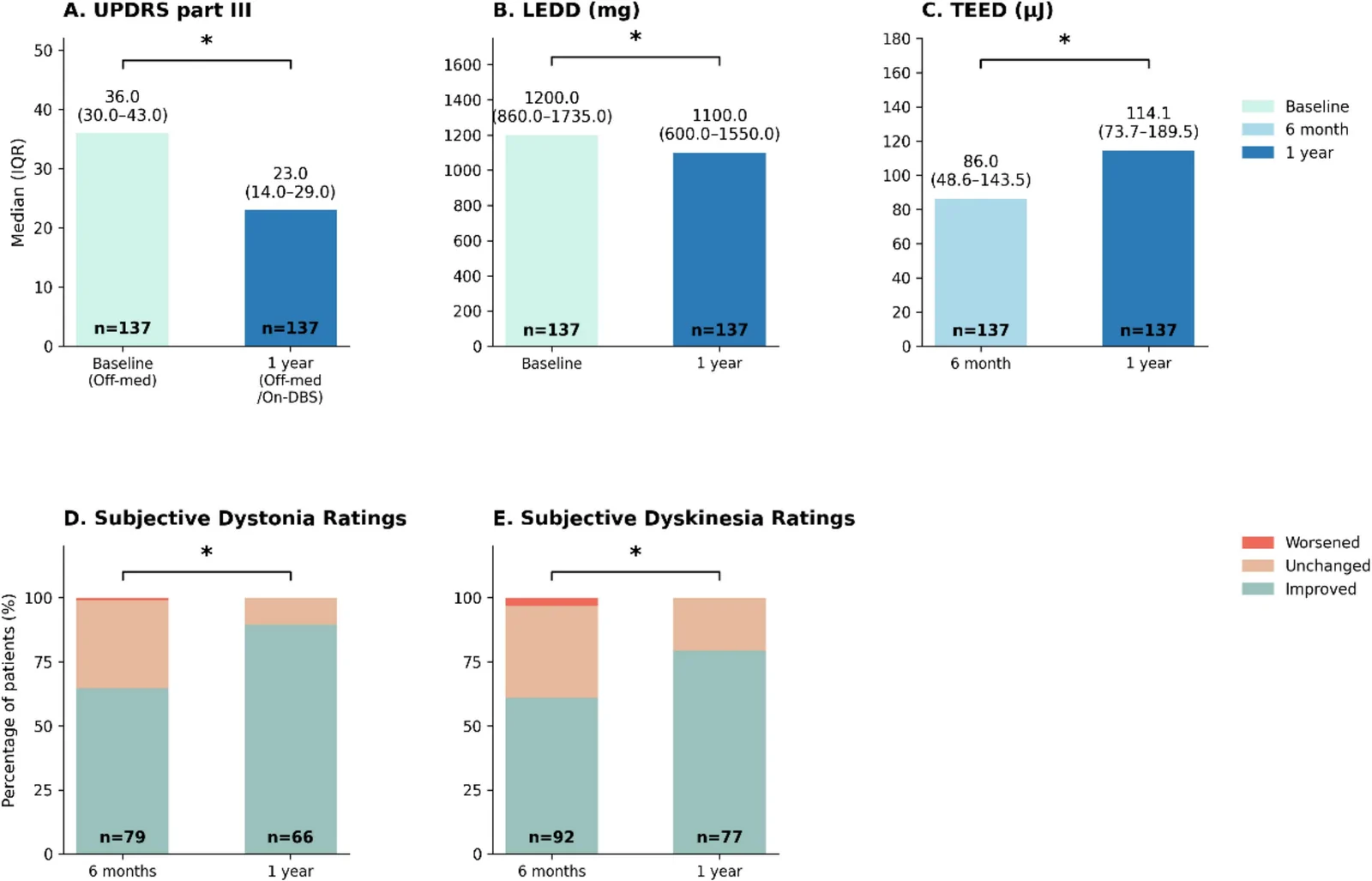
Longitudinal Changes in Motor Function, Medication, Stimulation Energy, and Subjective Symptom Ratings After GPi-DBS. A. Pre- and postoperative changes in the Unified Parkinson's Disease Rating Scale (UPDRS) Part III scores, assessed in a medication state that varied by center and timepoint (see Methods), showing significant improvement from baseline to 1 year postoperatively (Wilcoxon signed-rank test, FDR-adjusted *p* < 0.001). B. Pre- and postoperative levodopa equivalent daily dose (LEDD), which significantly decreased after surgery (FDR-adjusted *p* = 0.005). C. Total electrical energy delivered (TEED) increased significantly from 6 months to 1 year (FDR-adjusted *p* < 0.001). D. Distribution of subjective dystonia ratings (improved, unchanged, worsened) at 6 months and 1 year postoperatively, showing a significant distributional shift toward improvement (Stuart–Maxwell test, χ^2^ = 12.57, FDR-adjusted *p* = 0.0031). E. Distribution of subjective dyskinesia ratings at 6 months and 1 year, demonstrating a significant shift toward improvement (χ^2^ = 10.31, FDR-adjusted *p* = 0.0058). Error bars and boxplots represent medians and interquartile ranges unless otherwise specified.

Although neuropsychiatric vulnerability was commonly cited as a contributing factor to GPi selection, outcomes in this subgroup were not qualitatively different from those without such concerns in regard to dyskinesia or dystonia response.

This study was intentionally limited in scope. We did not attempt to compare GPi and STN outcomes, quantify effect sizes, or establish predictors of response. Rather, our goal was to reflect on how experienced multidisciplinary DBS teams apply existing evidence to individual patients and how they perceive outcomes in routine clinical care. The reliance on subjective assessments and retrospective documentation can introduce potential bias. Outcome classification was performed by the treating clinical team using a shared framework rather than by independent raters, and formal inter-rater reliability was not assessed; this is acknowledged as a limitation given the study's retrospective, two-center design.

In summary, across two academic centers, GPi-DBS was most selected for PD patients with prominent dyskinesia and dystonia, frequently in the context of neuropsychiatric vulnerability, although outcomes did not differ qualitatively by neuropsychiatric status. Clinicians consistently perceived sustained improvement in these symptoms at one year. These findings do not support comparative claims relative to STN-DBS; rather, they reflect real-world target-selection practice and clinician-perceived outcomes at two centers and highlight the continued clinical relevance of GPi-DBS in contemporary PD management.

## Financial disclosures for the previous 12 months

All authors completed the conflict-of-interest disclosure form per the journals requirements.

## Data access and responsibility

The investigators had full access to all study data and took responsibility for data integrity and accuracy of analysis.

## Ethical compliance statement

This study was approved by the institutional review board of Ethics Committee of the New York University Langone (approval number: S21-01610) and the University of Florida (approval number: 20150116). Written informed consent was obtained from all participants prior to their inclusion in the study. We confirm that we have read the Journal's position on issues involved in ethical publication and affirm that this work is consistent with those guidelines.

## Declaration of generative AI and AI-assisted technologies in the manuscript preparation process

The authors used ChatGPT (OpenAI) for language editing, formatting, translation from Japanese to English, and reference style conversion. No generative artificial intelligence tool was used to create original scientific content, analyze data, or influence study conclusions. All content was reviewed and approved by the authors, who take full responsibility for the manuscript.

## CRediT authorship contribution statement

**Nestor Beltre:** Writing – original draft, Methodology, Formal analysis, Data curation, Conceptualization. **Hikaru Kamo:** Writing – review & editing, Writing – original draft, Methodology, Formal analysis, Data curation, Conceptualization. **Gabriele Bellini:** Methodology, Formal analysis, Data curation, Conceptualization. **Ilona Cazorla-Morales:** Data curation, Conceptualization. **Adolfo Ramirez-Zamora:** Writing – review & editing, Supervision. **Michael S. Okun:** Writing – review & editing, Supervision. **Coralie de Hemptinne:** Writing – review & editing, Supervision. **Alon Mogilner:** Writing – review & editing, Supervision, Conceptualization. **Michael Pourfar:** Writing – review & editing, Supervision, Investigation, Formal analysis, Data curation, Conceptualization.

## Funding

The authors declare that there are no funding sources or conflicts of interest relevant to this work. A disclosure form is included in this submission.

## Declaration of competing interest

The authors declare the following financial interests/personal relationships which may be considered as potential competing interests: Hikaru Kamo reports a relationship with Japan Society for the Promotion of Science that includes: funding grants. Hikaru Kamo reports a relationship with Parkinson's Foundation Inc. that includes: funding grants. Michael S Okun reports a relationship with National Institute of Health that includes: funding grants. Michael S Okun reports a relationship with Parkinson's Foundation Inc. that includes: consulting or advisory and funding grants. Michael S Okun reports a relationship with The Michael J Fox Foundation that includes: funding grants. Michael S Okun reports a relationship with Parkinson Alliance that includes: funding grants. Michael S Okun reports a relationship with Frances C and William P Smallwood Foundation that includes: funding grants. Michael S Okun reports a relationship with Tourette Association of America that includes: funding grants. Michael S Okun reports a relationship with University of Florida Foundation that includes: funding grants. Adolfo Ramirez-Zamora reports a relationship with Medtronic Inc. that includes: consulting or advisory and speaking and lecture fees. Adolfo Ramirez-Zamora reports a relationship with Boston Scientific Corporation that includes: consulting or advisory. Adolfo Ramirez-Zamora reports a relationship with AbbVie Inc. that includes: consulting or advisory. Adolfo Ramirez-Zamora reports a relationship with BlueRock Therapeutics ULC that includes: consulting or advisory. Adolfo Ramirez-Zamora reports a relationship with Guidepoint that includes: consulting or advisory. Adolfo Ramirez-Zamora reports a relationship with Iota Inc. that includes: consulting or advisory. Alon Mogilner reports a relationship with Medtronic Inc. that includes: consulting or advisory. Michael Pourfar reports a relationship with Boston Scientific Corporation that includes: funding grants. Michael Pourfar reports a relationship with Abbott Laboratories Inc. that includes: consulting or advisory and speaking and lecture fees. In addition to prior disclosed relationships, Dr. Okun has the following financial disclosures. Royalties Hachette Book Group Demos Manson Amazon Smashwords Books4Patients Perseus Robert Rose Oxford University Press Cambridge University Press.

Educational / CME Activities WebMD / Medscape RMEI Medical Education American Academy of Neurology Movement Disorders Society Mediflix Vanderbilt University.

Editorial Roles NEJM Journal Watch Neurology JAMA Neurology If there are other authors, they declare that they have no known competing financial interests or personal relationships that could have appeared to influence the work reported in this paper.

## References

[1] Okun M.S. (2012). Deep-brain stimulation for Parkinson's disease. N. Engl. J. Med..

[2] Wong J.K., Deuschl G., Wolke R., Bergman H., Muthuraman M., Groppa S., Sheth S.A., Bronte-Stewart H.M., Wilkins K.B., Petrucci M.N., Lambert E., Kehnemouyi Y., Starr P.A., Little S., Anso J., Gilron R., Poree L., Kalamangalam G.P., Worrell G.A., Miller K.J., Schiff N.D., Butson C.R., Henderson J.M., Judy J.W., Ramirez-Zamora A., Foote K.D., Silburn P.A., Li L., Oyama G., Kamo H., Sekimoto S., Hattori N., Giordano J.J., DiEuliis D., Shook J.R., oughtery D.D.D., Widge A.S., Mayberg H.S., Cha J., Choi K., Heisig S., Obatusin M., Opri E., Kaufman S.B., Shirvalkar P., Rozell C.J., Alagapan S., Raike R.S., Bokil H., Green D., Okun M.S. (2022). Proceedings of the ninth annual deep brain stimulation think tank: advances in cutting edge technologies, artificial intelligence, neuromodulation, neuroethics, pain, interventional psychiatry, epilepsy, and traumatic brain injury. Front. Hum. Neurosci..

[3] Starr P.A., Shivacharan R.S., Goldberg E., Tröster A.I., House P.A., Giroux M.L., Hebb A.O., Whiting D.M., Leichliter T.A., Ostrem J.L., Metman L.V., Sani S., Karl J.A., Siddiqui M.S., Tatter S.B., Haq I.U., Machado A.G., Gostkowski M., Tagliati M., Mamelak A.N., Okun M.S., Foote K.D., Moguel-Cobos G., Ponce F.A., Pahwa R., Lyons K., Buetefisch C.M., Gross R.E., Luca C.C., Jagid J.R., Revuelta G.J., Takacs I., Pourfar M.H., Mogilner A.Y., Duker A.P., Mandybur G.T., Rosenow J.M., Zadikoff C., Khandhar S.M., Sedrak M., Phibbs F.T., Neimat J., Durphy J., Ramirez-Zamora A., Pilitsis J.G., Uitti R.J., Wharen R., Park M.C., Vitek J.L. (2025). Five-year outcomes from deep brain stimulation of the subthalamic nucleus for Parkinson disease. JAMA Neurol..

[4] Rughani A., Schwalb J.M., Sidiropoulos C., Pilitsis J., Ramirez-Zamora A., Sweet J.A., Mittal S., Espay A.J., Martinez J.G., Abosch A., Eskandar E., Gross R., Alterman R., Hamani C. (2018). Congress of Neurological Surgeons systematic review and evidence-based guideline on subthalamic nucleus and Globus pallidus internus deep brain stimulation for the treatment of patients with Parkinson's disease: executive summary. Neurosurgery.

[5] Ramirez-Zamora A., Ostrem J.L. (2018). Globus pallidus interna or subthalamic nucleus deep brain stimulation for Parkinson disease: a review. JAMA Neurol..

[6] Meng F., Cen S., Yi Z., Li W., Cai G., Wang F., Quintin S.S., Hey G.E., Hernandez J.S., Han C., Fan S., Gao Y., Song Z., Yi J., Wang K., Zhang L., Ramirez-Zamora A., Zhang J. (2022). Retrospective multicenter study on outcome measurement for dyskinesia improvement in Parkinson's disease patients with pallidal and subthalamic nucleus deep brain stimulation. Brain Sci..

