# Supplementary material for: Pallidal deep brain stimulation for Parkinson's disease: indications for target selection and perceived outcomes at two centers
Source: Clin Park Relat Disord. 2026 Oct 14;15:100513. doi: 10.1016/j.prdoa.2026.100513 (PMC13756181; doi:10.1016/j.prdoa.2026.100513)
Supplement: Supplementary file 1 [file mmc1.docx]

**Supplementary Appendix S1: Assessment Conditions by Center and Timepoint**

UPDRS Part III was assessed on-medication/on-stimulation at both centers preoperatively and at 6 months postoperatively. At the 1-year follow-up, assessment condition diverged by center: Center 1 continued to assess on-medication/on-stimulation, while Center 2 assessed off-medication/on-stimulation, consistent with each center's standard postoperative testing protocol. Because these conditions are not directly comparable, 1-year UPDRS Part III values are reported separately by center (Table 2) rather than pooled as a single value.

| Timepoint | Condition | Center 1 | Center 2 |
| --- | --- | --- | --- |
| Baseline | On-medication | 21.0 (15.0–30.0) | 21.0 (15.0–30.0) |
| 6 months | On-medication/on-stimulation | 23.0 (16.2–28.8) | 19.0 (12.0–26.0) |
| 1 year | On-medication/on-stimulation | 23.5 (16.2–30.8) | — |
| 1 year | Off-medication/on-stimulation | — | 23.0 (14.0–29.0) |

**Supplementary Appendix S2: Statistical Methods**

All statistical analyses were conducted using R (version 4.4.2; R Foundation for Statistical Computing, Vienna, Austria). Continuous variables are presented as median (interquartile range, IQR). Normality was assessed using the Shapiro–Wilk test; as most variables were non-normally distributed, nonparametric methods were used throughout.

**Paired and unpaired comparisons.** Paired continuous outcomes (UPDRS Part III, levodopa-equivalent daily dose [LEDD], total electrical energy delivered [TEED]) were compared using the Wilcoxon signed-rank test. Unpaired between-group comparisons (e.g., patients with vs. without neuropsychiatric indications) used the Wilcoxon rank-sum test for continuous variables and Fisher's exact test for categorical variables. Paired categorical outcomes (subjective dystonia and dyskinesia ratings, 6 months vs. 1 year) were compared using the Stuart–Maxwell test for marginal homogeneity.

**Multiple comparisons.** All p-values were adjusted for multiple comparisons using the Benjamini–Hochberg false discovery rate (FDR) procedure. Significance was set at p < 0.05 (FDR-adjusted).

**Predictors of outcome.** For the 1-year dystonia and dyskinesia outcomes, ratings were dichotomized as improved vs. not improved (no patients were rated worsened at 1 year) and entered into separate binary logistic regression models, each with GPi-DBS indication (dystonia, dyskinesia, neuropsychiatric), sex, age, preoperative on-medication UPDRS Part III, and preoperative LEDD as predictors. Odds ratios (95% CI), Nagelkerke's pseudo-R², and area under the curve (AUC) were calculated for each model:

- *Dystonia improvement model:* Nagelkerke's R² = 0.314, AUC = 0.804. No predictor reached statistical significance; GPi selection for dystonia showed OR = 0.36 (95% CI 0.03–4.16, p = 0.40).
- *Dyskinesia improvement model:* Nagelkerke's R² = 0.191, AUC = 0.719. GPi selection for dystonia was significantly associated with dyskinesia improvement (OR = 7.65, 95% CI 1.53–64.69, p = 0.028); no other predictor was significant.

**Missing data.** Patients missing a given follow-up timepoint were excluded from that specific comparison (pairwise deletion) rather than imputed, and were retained in any other analysis for which their data were complete.

**Sample sizes per comparison:**

| **Outcome** | **Timepoint** | **n assessed** |
| --- | --- | --- |
| Subjective dystonia rating | 6 months | 79 (51 improved + 27 unchanged + 1 worsened) |
| Subjective dystonia rating | 1 year | 66 (59 improved + 7 unchanged + 0 worsened) |
| Subjective dyskinesia rating | 6 months | 92 (56 improved + 33 unchanged + 3 worsened) |
| Subjective dyskinesia rating | 1 year | 77 (61 improved + 16 unchanged + 0 worsened) |

**Supplementary Appendix S3: Examples of qualifying language, by category:**

Improved — Dyskinesia:

- "He has not experienced any return of dyskinesias"
- "Significant improvement...particularly with regards to dyskinesias"
- "No dyskinesias so they feel DBS has been very helpful"

Unchanged — Dystonia:

- "GPi selected for camptocormia which did not respond to stimulation"

**Supplementary Appendix S4:** **Table 1. Baseline characteristics of patients with PD who underwent GPi-DBS**

| Variable | All | Site A | Site B |
| --- | --- | --- | --- |
| Total (n) | 137 | 42 | 95 |
| Gender (Female/Male) | 48/89 | 17/25 | 31/64 |
| Race |  |  |  |
| Non-Latino Caucasians | 111 | 23 | 88 |
| Latino | 5 | 5 | 0 |
| Black | 2 | 1 | 1 |
| Asian | 6 | 6 | 0 |
| Other or not documented | 19 | 12 | 7 |
| Age at onset (years) | 53.0 (45.0–60.0) | 53.0 (47.2–59.8) | 53.0 (45.0–60.0) |
| Age at surgery (years) | 65.0 (57.0–71.0) | 64.0 (58.0–70.8) | 65.0 (56.0–71.0) |
| Disease duration (years) | 9.0 (7.0–13.0) | 10.0 (7.0–13.8) | 9.0 (7.0–13.0) |
| UPDRS III (ON) | 21.0 (15.0–30.0) | 24.5 (16.0–34.8) | 21.0 (15.0–28.0) |
| LEDD (mg) | 1200.0 (860.0–1735.0) | 1177.0 (750.0–1623.0) | 1275.0 (870.0–1797.8) |
| Reasons for GPi selection |  |  |  |
| Dystonia | 67 (48.9%) | 7 (16.7%) | 60 (63.2%) |
| Dyskinesia | 103 (75.2%) | 34 (81.0%) | 69 (72.6%) |
| Neuropsychiatric symptoms | 42 (30.7%) | 11 (26.2%) | 31 (32.6%) |

Values are presented as median (interquartile range). Multiple reasons could be assigned per patient. DBS, deep brain stimulation; GPi, globus pallidus internus; UPDRS, Unified Parkinson’s Disease Rating Scale; LEDD, levodopa equivalent daily dose.

Note: Site-level differences in the distribution of GPi selection indications (e.g., dystonia as the reported rationale in 16.7% of Site A patients versus 63.2% of Site B patients) likely reflect differing referral patterns and clinical practice between centers rather than differences in patient population; postoperative outcomes were nonetheless directionally consistent across sites (see Results).
